# Entropy-Driven Porous Liquids Allowing Gas Solubility in Solvent-Filled Imine-Based Porous Organic Cages

**DOI:** 10.1021/acs.jpcb.5c04176

**Published:** 2025-08-06

**Authors:** Chao-Wen Chang, David S. Sholl

**Affiliations:** † School of Chemical & Biomolecular Engineering, 1372 Georgia Institute of Technology, Atlanta, Georgia 30332, United States; ‡ 6146 Oak Ridge National Laboratory, Oak Ridge, Tennessee 37839, United States

## Abstract

Porous liquids offer a promising platform for gas separation by combining fluid processability with intrinsic molecular porosity. Traditional Type II porous liquids are formed by dissolving porous molecular cages in size-excluded solvents, limiting solvent options and practical applications. In this work, we introduce a novel method of creating Type II porous liquids using common small solvents, where intrinsic porosity is achieved at elevated pressures due to the selective displacement of solvent molecules by gas molecules within the cage structures. Using molecular simulations, we investigate the behavior of CO_2_ in solutions of the imine-based porous organic cage CC13 dissolved in small molecular solvents such as chloroform and 1,2-dimethoxyethane (DME). Grand canonical Monte Carlo simulations of solid-state CC13 reveal that selectivity reversal, where smaller CO_2_ molecules displace larger solvent molecules inside the cage, is achievable at sufficiently high pressures. Temperature quench molecular dynamics simulations confirm that while CO_2_ displacement within chloroform-filled cages is limited, DME enables entropy-driven cage CO_2_ occupancy at pressures as low as ∼23 bar, setting up the foundation of an alternative way of forming Type II porous liquids.

## Graphical abstract

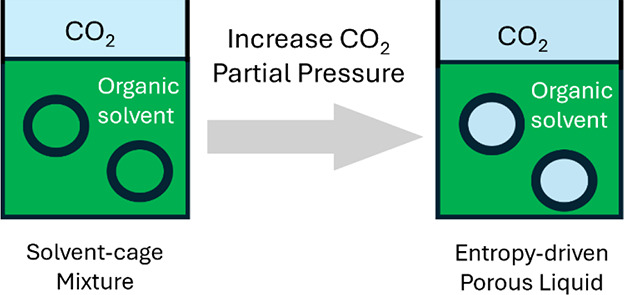

## Introduction

Porous liquids combine the ease of manipulation of liquids with the advantages of porosity in solids, including size-selectivity and capacity for molecular guests.^1,2^ As a result, porous liquids offer distinctive benefits for gas storage and separation processes. The concept of a liquid with inherent, stable porosity was initially introduced by James and co-workers in 2007.^3^ Since then, three categories of porous liquids have been identified. Type I porous liquids consist of molecular species with intrinsic porosity.^4–6^ Type II features a size-excluded solvent solubilizing zero-dimensional pore carriers, providing permanent intrinsic porosity. Example molecules as cavity hosts for Type II porous liquids include imine-modified porous organic cages (POCs) or metal–organic cages (MOCs) dissolved in bulky organic solvents.^7–12^ Type III porous liquids are dispersions of microporous solids in cavity-excluded liquids. Solutes that have been explored for Type III porous liquids include MOF or zeolite nanoparticles dispersed in bulky solvents like ionic liquids, PDMS, and 2′-hydroxyacetophenone.^13–17^

The Type II and Type III porous liquids described above are conventionally developed by selecting solvents defined by bulky molecules, ensuring that they are too large to penetrate the porous molecular structures. However, many of these potential bulky solvents may not be good solvents, limiting the selection of solvent/solute combinations that make effective porous liquids.^18^ In this paper, we introduce a variant of Type II porous liquids that broadens the scope of potential solvents by using entropy-driven effects to access intrinsic porosity in cage molecules that are initially dissolved in a solvent that is small enough to fill the cages. This situation was motivated by the entropic adsorption effects that are well-known in porous solids for mixtures of adsorbates of different sizes.^19–25^ If a porous solid is exposed to a mixture of a larger molecule with a higher adsorption affinity and a smaller molecule with a lower adsorption affinity, the pores will predominately fill with the larger molecule at low pressures (or fugacities). At sufficiently high pressures or fugacities, however, entropic contributions to the free energy of the system favor adsorption of the smaller molecule because more molecules can fit into the pores, leading to partial or complete exclusion of the larger molecule from the pores.^19,20^

Below, we explore the possibility of accessing entropy-driven porosity in a liquid containing POCs that have no effective porosity at low pressures. We focus for this purpose on liquids formed by dissolving a prototypical [4+6] imine-based POC, CC13,^26–28^ in readily available solvents. For example, several previous studies have demonstrated chloroform as an effective solvent for POCs.^18,27,29^ Chloroform has previously been considered infeasible as a solvent for making porous liquids because it is small enough to enter the interior of POCs, thus removing the cages’ porous character. We use molecular simulations to explore the absorption of CO_2_ in liquids composed of selected solvents and CC13. At low CO_2_ pressures, absorption occurs only in the solvent phase, so the inclusion of POCs imparts no porous character to the liquid. At sufficiently high pressures, however, entropic effects displace the solvent from the porous cages, producing a porous liquid with distinctive binding sites for the gas molecule inside the porous cages and in the solvent. These observations expand the range of solvent/cage combinations that can be considered when developing liquids with intrinsic porosity.^30,31^

## Computational Methods

To simulate CC13 in either the solid state or as individual cages in a solvent, we employed the OPLS-AA forcefield^30^ to describe the inter- and intramolecular interactions. The TraPPE force field^31^ was utilized for CO_2_, *n*-pentane, neo-pentane, acetone, isopropyl alcohol (IPA), 1,2-dimethoxyethane (DME), tetrahydrofuran (THF), and ethyl acrylate. The force field parameters for chloroform were adopted from the work of Kamath et al.^32^ GAFF was used as a force field for dichloromethane (DCM) and *N*,*N*-dimethylformamide (DMF).^33^ Vapor–liquid equilibrium data for chloroform and DCM were validated in Figure S1. In all cases, we used Lorentz–Berthelot combining rules to describe interactions between atoms in cages and solvent or gas molecules. All of our MD simulations were carried out using the LAMMPS software. For all MD simulations, a time step of 0.5 fs was used, periodic boundary conditions were applied in all directions, and a nonbonded interaction cutoff of 12 Å was used. Ewald summation was used to compute long-range Coulombic interactions. A Nosé–Hoover thermostat and a Nosé–Hoover barostat with damping coefficients of 100 and 1000, respectively, were applied to regulate the temperature and pressure.

### Crystalline CC13α CO_2_ and Solvent Isotherms

To gain initial insight into how molecules occupy the intrinsic porosity of CC13 cages, we performed simulations of the CC13α solid using a crystal structure that was energy-minimized from the original report of Hasell et al.^26^ The energy-minimized structure was obtained by density functional theory using the VASP^34^ software using the PBE functional with D3 dispersion corrections in calculations that sampled reciprocal space only at the Γ point with an energy cutoff of 400 eV. Because our aim with these calculations was to obtain qualitative insight rather than quantitative predictions, we performed simulations that assumed the CC13 cages in the solid crystal were rigid during adsorption. We performed simulations for both single-component adsorption and equimolar binary mixture adsorption by grand canonical Monte Carlo (GCMC) using RASPA.^35^ Simulation details are stated in our previous work.^15,36^ Simulations were performed using 10^5^ Monte Carlo cycles for equilibrium and 10^6^ Monte Carlo cycles for data collection, with each Monte Carlo cycle including translation, rotation, reinsertion, and (for mixtures) identity swap moves with equal probability. Preliminary tests indicated that these choices gave well-converged results.

### CO_2_ Solubility Predictions in Pure Solvents and the 10 wt % Solvent-CC13

Temperature quench molecular dynamics (TQMD) is an effective technique for determining fluid phase equilibria that involves suddenly quenching a one-phase fluid system to a lower temperature, causing the formation of coexisting phases that quickly reach equilibrium.^37,38^ Local densities and compositions can then be determined by analyzing the coexisting domains. We demonstrated the use of TQMD simulations for multiple-component systems including gas absorption in Type II porous liquids previously.^15,36^ For this work, chloroform, DME, and neo-pentane were chosen as solvents. To carry out the simulation of CO_2_ absorption in neat solvents, we randomly placed varying numbers of CO_2_ molecules (50, 100, 200, 300) and a fixed number of solvent molecules (600) in a fixed-sized cubic box with edges of 200 Å, with insertions made only if atoms in the inserted molecules had distances of at least 2 Å from all existing atoms. Likewise, to simulate CO_2_ absorption in a liquid with 10 wt % CC13 dispersed in the solvent, we randomly placed varying amounts of CO_2_ molecules (100, 200, 400, 600, 800, 1000, 1200), a fixed number of CC13 cages (10), and an appropriate number of solvent molecules (to maintain the 10 wt % composition) within the same simulation box dimensions. All systems were initially energy-minimized to remove unfavorable steric interactions. We then gradually reshaped the simulation box into 40 × 40 × 400 Å (or 40 × 40 × 160 Å for a solvent-gas system) rectangular dimensions during a 150 ps *NVT* simulation at 600 K. After raising the temperature to 900 K for 1 ns to ensure all components are well-mixed, we quenched the system to the target temperature (273 or 300 K) in a single time step. We then ran a canonical ensemble (*NVT*) simulation at the target temperature for 10 ns, which typically leads to the formation of stable, locally equilibrated liquid and vapor phases. To minimize the liquid’s surface tension, the vapor–liquid boundaries consistently aligned parallel to the box’s *x*–*y* plane (*x* and *y* are the short edges of the simulation volume). Configurations were recorded every 25 ps during the *NVT* ensemble simulations. The solubility of CO_2_ was calculated by measuring its concentration in the liquid bulk phase and using the ideal gas law to determine the pressure based on the CO_2_ density in the gas phase. The density profile of CO_2_ within the simulation box was analyzed along the *z*-direction. Density values were recorded at points where the density curve reached a plateau within the liquid and gas phases.^36^

### Spatial Analysis of 10 wt % Chloroform-CC13 Liquid Phases

TQMD is well suited to establishing vapor–liquid equilibrium conditions, but it is convenient to directly perform simulations of the liquid phase to allow for further analysis. To this end, we performed isothermal–isobaric (*NPT*) MD of simulation volumes containing only the liquid phase. The number of CO_2_ molecules for these simulations was determined based on TQMD-calculated solubilities and pressures. Specifically, we employed an expanded system comprising 40 CC13 cages, 2880 chloroform molecules, and varying quantities of CO_2_ (228, 472, 860, 1256, 2036, and 2940, corresponding to pressures of 7, 12, 22, 31, 39, and 46 bar, respectively) in a cubic box with edges of 200 Å. These structures were then relaxed by isothermal–isobaric (*NPT*) MD for 10 ns, applying isotropic stresses to each dimension, followed by a canonical (*NVT*) MD production run for 10 ns, all at 300 K. The configurations were recorded every 25 ps during the *NVT* ensemble simulations.

### Definition of CO_2_-Rich and CO_2_-Lean Phases

The CO_2_-rich and CO_2_-lean phases are defined based on the spatial location of CO_2_ molecules relative to the CC13 cage. Following our previous work,^36^ we use the radial distribution function of solvated CC13 cages to establish spatial boundaries. Regions within 4 Å from the center of mass (COM) of a cage are considered inside the cage; distances between 4 and 9 Å represent the cage interface; and regions beyond 9 Å are regarded as outside the cage. Using this framework, we define the CO_2_-rich phase as the region containing CC13 cages, while the CO_2_-lean phase corresponds to the surrounding bulk liquid region.

## Results

### Binary Adsorption in Crystalline CC13α

We know that chloroform is a good solvent for imine-based POCs and outperforms many solvents in terms of solubility.^18^ To gain initial insight into the coadsorption of chloroform and CO_2_ in CC13 cages, we performed calculations for the rigid crystalline form of CC13α using GCMC simulations. Structurally, CC13α presents two distinct types of cavities: the intramolecular cavity within each cage and the intermolecular interstices between cages. Although CO_2_ can occupy the interstitial spaces between CC13 cages in addition to the internal cavities, this configuration still provides insight into the interactions among CC13 cages, chloroform, and CO_2_. Swelling or deformation of crystalline cages can be important in making precise predictions about adsorption in these solid phases, but these effects are replaced by solvent effects in porous liquids. For this reason, we approximated the solid crystals as rigid in these initial calculations.

Single-component adsorption for CO_2_ and chloroform in crystalline CC13α was simulated at 273, 300, and 325 K, as shown in Figure 1a. Fugacity was determined by the Peng–Robinson equation of state, as shown in Figure S2. Unsurprisingly, a lower temperature causes the onset of gas loading to occur at a lower pressure. Chloroform isotherms exhibit two distinct step changes, indicating that adsorption occurs in two stages. The first step is associated with chloroform molecules filling the cages, as these in-cage pockets are significantly more favorable than the interstitial sites due to their bulky size.

**Figure 1:**
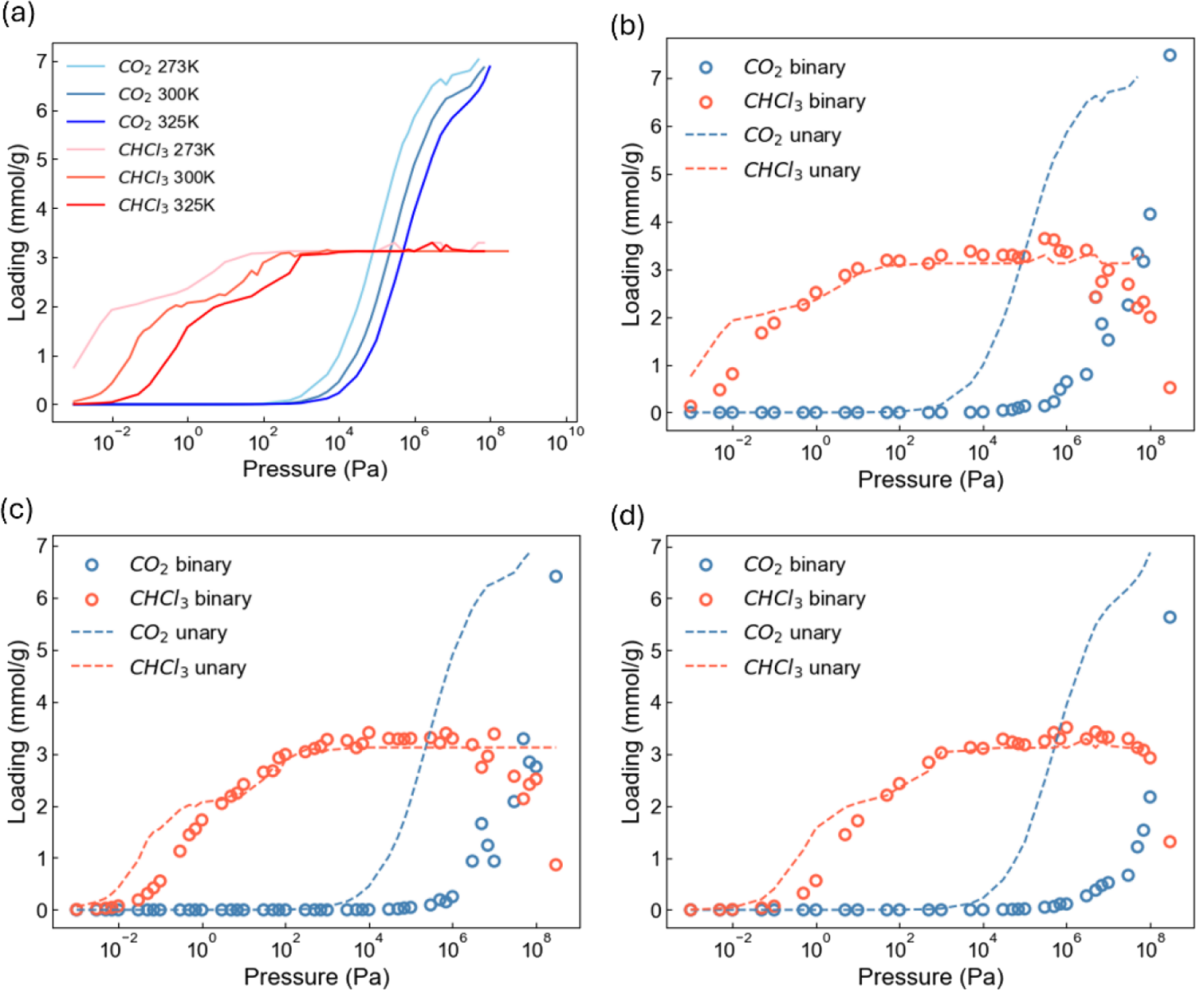
(a) GCMC data for single-component adsorption isotherms of CO_2_ and chloroform in crystalline CC13α at 273, 300, and 325 K. (b–d) GCMC data for binary-component adsorption isotherms of CO_2_ and chloroform in the CC13α crystal at (b) 273, (c) 300, and (d) 325 K. Dashed lines represent the single-component isotherms of CO_2_ and chloroform.

Binary adsorption for equimolar CO_2_ and chloroform in crystalline CC13α was simulated at 273, 300, and 325 K, as shown in Figure 1b–d. At low pressures, CC13 favors chloroform over CO_2_ because chloroform has greater binding strength, as would be expected from its larger molecular weight. Selectivity reversal is observed at pressures of 355, 360, and 1296 bar for temperatures of 273, 300, and 325 K, respectively, and above these pressures, CO_2_ adsorption is favored in the cages. This phenomenon arises from the superior packing efficiency of smaller CO_2_ molecules under high loading conditions. The concave curves observed in the chloroform mixture isotherms are attributed to entropic effects.^23^ Lower temperatures allow this selectivity reversal to happen at lower pressures.

Krishna suggested that to facilitate selectivity reversal by entropic effects in porous materials, (1) the difference in packing efficiency, i.e., saturation capacities between two species, should be increased while (2) reducing the difference in binding strength.^19^ To examine whether this can be accomplished by varying the solvent for CC13, we performed similar simulations for additional common solvents. Specifically, we studied DCM, neo-pentane, *n*-pentane, acetone, IPA, DMF, DME, THF, and ethyl acrylate. All simulations were carried out at 273 K to keep the chosen solvents in the liquid phase. Properties obtained from the single-component isotherm are listed in Table 1 (also see Figure S4). In this rigid crystal structure, the CO_2_ saturation loading is 8.3 molecule/cage, a loading that includes CO_2_ molecules located both within the cage and in the interstitial regions. At 0.001 Pa, the heat of adsorption for CO_2_ from our simulations, *Q*_st_, is −27.0 kJ/mol. The solvents are listed in Table 1 from the lowest pressure of selectivity reversal (neo-pentane) to the highest pressure (acetone).

**Table 1:** Pressure of Selectivity Reversal from Binary-Component Adsorption for Equimolar CO_2_ and 10 Solvent Species in CC13α at 273 K^a^ ^,^ ^b^

solvent species	*q*_sat_^solvent^ – *q*_sat_^CO_2_^ (molecule/cage)	*Q*_st_^solvent^ – *Q*_st_^CO_2_^ (kJ/mol)	pressure of selectivity reversal (bar)
neo-pentane	–7.3	43.0	1
ethyl acrylate	–5.7	92.6	4
1,2-dimethoxyethane	–5.5	53.6	26
tetrahydrofuran	–5.3	55.2	32
*n*-pentane	–5.6	41.2	43
*N*,*N*-dimethylformamide	–5.0	30.2	316
chloroform	–5.3	52.2	355
isopropyl alcohol	–4.3	49.3	608
dichloromethane	–4.2	48.4	1603
acetone	–4.3	52.7	1787

a Obtained from single-component adsorption of solvent and CO_2_ in CC13α at 273 K.

b The difference in saturation loading *q*_sat_ and enthalpy of adsorption *Q*_st_^0^ at 0.001 Pa between each solvent and CO_2_ is also shown.

As might be expected, the pressure of selectivity reversal increases as the differences between CO_2_ and solvent saturation loading decrease. There is not a similar simple trend associated with the differences in the CO_2_ and solvent heats of adsorption. In this rigid model of a CC13 crystal, saturation loading differences >5.5 molecules/cage lead to selectivity reversal at pressures below 40 bar, conditions more feasible in real-world applications, whereas there is no obvious trend for differences in the heat of adsorption. We qualitatively conclude from Table 1 that the compactness of the molecular conformations exerts a greater influence on selectivity reversal than does the binding strength. We performed linear regression using Krishna’s two criteria to correlate the selectivity reversal pressure (Figure S6). This analysis points to the same conclusion that the difference in saturation loading exerts a stronger influence on the reversal pressure than the difference in the heat of adsorption.

### CO_2_ Absorption in Solvent-CC13 Liquids

GCMC models have limitations, particularly in their inability to account for cage flexibility and the dynamic, time-dependent process of guest molecule encapsulation. To address this, we employed solvated systems with fully flexible cage representations by using molecular dynamics. We selected three solvents, namely, chloroform, DME, and neo-pentane, for further examination of the possibility of forming entropy-driven porous liquids. Based on our previous work, chloroform and DME are good solvents for dissolving the CC13 crystal.^18^

First, we examined the absorption of CO_2_ in liquids made from CC13 cages dissolved in chloroform under pressures relevant for laboratory experiments. We performed TQMD to simulate CO_2_ solubility in the pure chloroform and a representative 10 wt % chloroform-CC13 liquid (see Figure 2a).

. Chloroform exchange between the interior of the cages and the surrounding solvent can be seen in our simulations (Figure S9), indicating that chloroform is not sterically restricted by the cage windows. This outcome reiterates the observation that chloroform fills the solvated cages.

Two methods were employed to deploy CC13-chloroform molecules and validate the robustness of the TQMD approach, as illustrated in Figure S10. In the first method, CC13 cages, chloroform, and CO_2_ molecules were randomly placed in the simulation box, followed by a temperature quench procedure under the canonical ensemble. The second method involved initially placing only CC13 cages and chloroform randomly in the simulation box, performing the same temperature quench procedure, allowing distinct liquid and gas phases to form, and then introducing CO_2_ into the gas phase. The system was subsequently equilibrated at 300 K in the canonical ensemble until equilibrium was reached. As observed in Figure S11, CO_2_ initially present in the gas phase gradually diffused into the liquid phase until reaching equilibrium. Method 2 more accurately reflects the real-world process of gas sorption in a liquid sorbent, while Method 1 offers a faster route to equilibrium. Both methods yield comparable CO_2_ loading for the 10 wt % chloroform–CC13 system, as shown in Figure S12, demonstrating the reliability and robustness of the TQMD approach.

We observed that for both simulation methods only a few CO_2_ molecules occupied the interior of the cages at pressures up to ∼46 bar (see Figure 3a), indicating that selectivity reversal had not yet occurred under these conditions. This is consistent with the expectation from Figure 1 that far higher pressures are required before CO_2_ can strongly displace chloroform from the CC13 cages. Moreover, from Figure 2a, the addition of CC13 cages does not enhance the CO_2_ loadings compared to pure chloroform. This is consistent with the observation that CO_2_ barely enters the cage. In Figure 3a, a lower temperature leads to a higher number of in-cage CO_2_ molecules. However, even at 273 K and 30 bar, the occupancy remains below one molecule of CO_2_ per cage. Therefore, our simulations suggest that the entropy-driven formation of the chloroform-CC13 porous liquid does not occur under these conditions. Pure CO_2_ liquefies at 67 bar at 300 K,^39^ indicating that the thermodynamic conditions of our TQMD simulation approach the threshold where CO_2_ is no longer a gas in the bulk phase.

**Figure 2:**
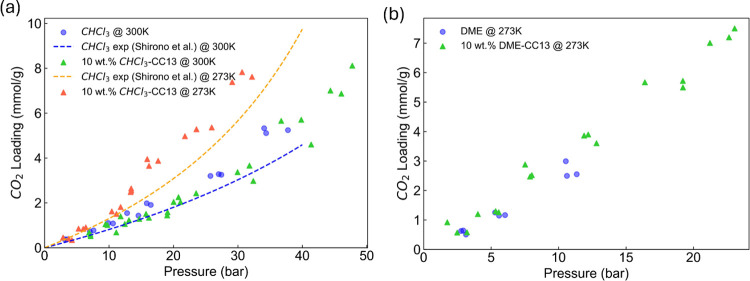
(a) CO_2_ solubilities in chloroform (blue circle) and 10 wt % chloroform-CC13 (triangles) obtained by TQMD at 300 and 273 K. The dashed lines are the CO_2_ uptake in pure chloroform using Henry’s law obtained by Shirono et al.^40^ Unlike solid sorbents where framework molecules cannot move freely, as the CO_2_ loading increases, CO_2_ displaces the liquid sorbent molecules, leading to a decrease in the solvent’s density. This results in a slightly convex CO_2_ sorption curve. (b) CO_2_ solubilities in DME (blue circle) and 10 wt % DME-CC13 at 273K.

**Figure 3:**
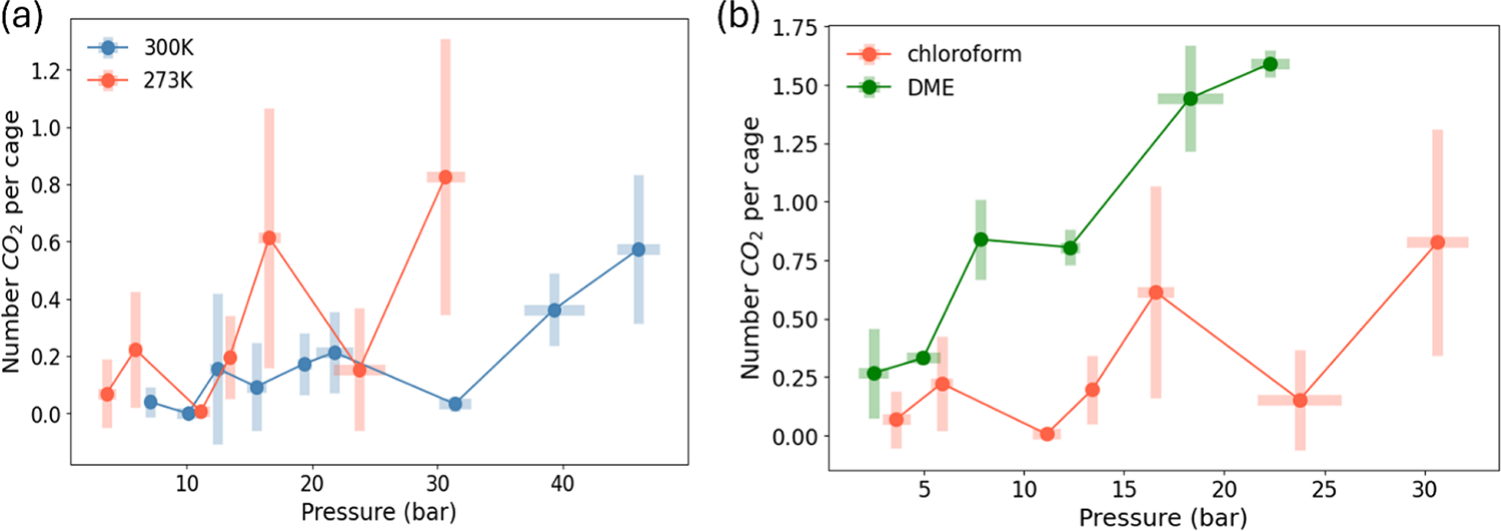
Number of in-cage CO_2_ as a function of CO_2_ partial pressure, comparing (a) the effect of temperature for 10 wt % chloroform-CC13 and (b) different solvents at 273 K.

Motivated by the binary GCMC results from solid CC13 crystals in Table 1, we studied the possibility of DME for forming entropy-driven porous liquids. In Figure 3b, the 10 wt.% DME-CC13 solution contains an average of 1.6 CO_2_ molecules per cage at a CO_2_ partial pressure of 22.9 bar. This observation demonstrates the feasibility of the entropy-driven porous liquid. Additional evidence can be seen in Figure 2b, where the inclusion of CC13 cages slightly increases the CO_2_ loading compared to that of pure DME.

Motivated by the results in Table 1, we also examined neo-pentane as a possible solvent for CC13. Neo-pentane turns out to be a poor choice for demonstrating entropy-driven porosity for multiple reasons. Although our GCMC simulations suggest that neo-pentane can occupy CC13 cages (one molecule per cage), these simulations cannot give information on potential kinetic limitations for solvent molecules filling the cages. In our TQMD simulations with neo-pentane, the bulky nature of neo-pentane prevented it from entering CC13 cages during the MD simulations we performed. As a result, the neo-pentane–CC13 mixtures we simulated contained intrinsic porosity at all pressures. In addition, neo-pentane is nonpolar and appears to be a poor solvent for CC13. This problem is indicated in Figure S13b, which shows that CO_2_ can enter the cage even at low pressures and the cage distribution in the solvent is localized.

### Spatial Analysis

The Gibbs phase rule indicates that a three-component system can support the coexistence of two liquid phases and one gas phase. Biphasic solvents are a well-characterized example of two liquid phases coexisting in this situation.^41–43^ We examined our simulation using 10 wt % chlroform-CC13 at 300 K to see whether there was any indication of formation of two liquid phases. This analysis also provides insight into whether POCs tend to aggregate under high-pressure conditions (e.g., 46 bar). As shown in Figure S14, the nonuniform and irregular density profiles suggest possible aggregation behavior.

We first discuss the distribution of CC13 cages in the bulk liquid phase using a larger simulation volume than those of the simulations discussed above. We applied isotropic pressure to a cubic simulation box containing four times the number of molecules as in the original 10 wt % chloroform–CC13 system to investigate the potential aggregation behavior of the cages. We calculated cages’ COM pair distances and obtained the radial distribution functions shown in Figure 4a at various pressures. As might be expected, CC13 has a tendency to be closer to each other at higher pressures (e.g., 39 and 46 bar). Density-based spatial clustering of applications with noise (DBSCAN)^44^ is a clustering algorithm that identifies clusters by grouping points that are closely packed together while marking points in low-density regions as outliers or noise, in this case, isolated cages. In DBSCAN, a core point must have a specified minimum number of neighboring points (two in our case) within a defined radius. Here, the radius is set to 14.3 Å, representing the distance between two cages, each with a radius of 4.5 Å and a distance threshold. This distance threshold ensures that a chloroform molecule with a dynamic radius of 5.3 Å cannot fit within the space between two adjacent cages, thereby defining the clustering boundary. This analysis shows that small clusters that consist of just 2–3 cages form more frequently at 39 and 46 bar than at lower pressures (see Table S1). This observation is consistent with the radial distribution function in Figure 4a.

**Figure 4:**
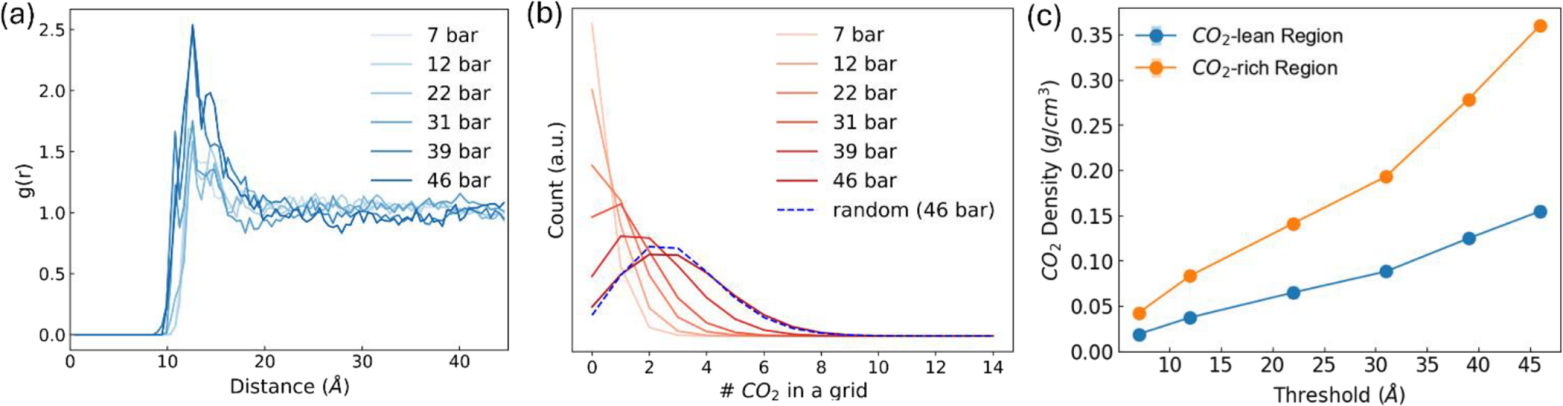
(a) Radial distribution function of the COM between two CC13 molecules at various pressures. (b) Histogram representing the frequency distribution of grid cells containing specific quantities of CO_2_ molecules. The simulation box was divided into a 10 × 10 × 10 mesh grid, and the number of CO_2_ molecules within each grid cell was counted to generate the histogram, illustrating the spatial distribution of CO_2_ across the grid. (c) CO_2_ density as a function of pressure for different regions of 10 wt % chloroform-CC13 at 300 K.

To analyze the spatial distribution of CO_2_, the simulation box from simulations at each pressure was divided into a 10 × 10 × 10 mesh grid. The number of CO_2_ molecules within each grid cell was counted, and a histogram was generated to illustrate the frequency of grid cells containing specific quantities of CO_2_. The resulting distribution is presented in Figure 4b. The spatial distribution of CO_2_ follows a near-Poisson distribution, suggesting that the CO_2_ molecules are approximately randomly distributed within the simulation volume, despite the presence of other components. For comparison, a completely random distribution is represented by the dashed blue line in Figure 4b. This distribution assumes the same number of CO_2_ molecules as the total loading at 46 bar. Thus, spatial analysis of both the CO_2_ and CC13 cages indicates the absence of distinct liquid–liquid phase separation or the formation of large cage clusters.

While a single phase is maintained in the bulk liquid with no phase separation or large cage clustering, subregional differences in the CO_2_ concentration are still observed. Specifically, we identified CO_2_-rich and CO_2_-lean regions attributed to chloroform’s higher affinity with the cage structure relative to CO_2_. To this end, a boundary was defined at a distance of 9 Å from the COM of the cages. CO_2_ molecules beyond 9 Å are considered to reside in the CO_2_-rich region, representing the bulk liquid phase, whereas those within 9 Å of the cage COM are classified as being in the CO_2_-lean region. Additional details of defining this boundary are available in our previous publication.^36^ There is a significant difference in the local CO_2_ concentration in these two regions, as shown in Figure 4c.

## Conclusions

We explored the potential for entropy-driven effects to enable porosity in liquids formed by dissolving imine-based POCs in small solvents like chloroform. Using molecular simulations, we demonstrated that while chloroform completely fills CC13 cages at low pressures, entropic effects at higher pressures (above 300 bar) displace the solvent, creating a pseudoporous environment suitable for CO_2_ adsorption. Lower temperatures are more conducive to this entropy-driven selectivity reversal, making them more favorable for the formation of entropy-driven Type II porous liquids.

Binary-component GCMC simulations of 10 organic solvents in crystalline CC13 offered insights into selecting an appropriate solvent for an entropy-driven Type II porous liquid. A useful solvent for this task requires a sufficiently bulky structure to reduce the configurational entropy inside CC13 cages. Among the ten solvents examined, five show mild selectivity reversal pressures for CO_2_ (<40 bar), making them plausible candidates for forming entropy-driven porous liquids.

TQMD simulations were used to assess the CO_2_ absorption behavior in organic liquids containing CC13 cages. Chloroform, though effective at dissolving CC13, allows solvent molecules to fill the cages even at high pressure, limiting CO_2_ uptake and preventing entropy-driven cage occupancy under experimentally relevant conditions. In contrast, DME, a bulkier solvent, enables higher in-cage CO_2_ loading (1.6 molecules per cage at ∼23 bar). The combination of DME and CC13 cages has no effective porosity for CO_2_ at low pressures but allows porosity-driven uptake of CO_2_ at experimentally achievable pressures. This is the first example of a Type II porous liquid with entropy-driven (and therefore, pressure-dependent) porosity. These results point to a new avenue for endowing liquids with intrinsic porosity and highlight the importance of solvent size and compatibility in the design of porous liquids to achieve this entropy-driven behavior.

Our selection of potential solvents for entropy-driven cage filling in Table 1 focused on the enthalpy of adsorption of the relevant species from the gas/vapor phase. This is a convenient quantity to use, because it allow qualitative comparisons with earlier work on entropy-driven selectivity in porous materials and GCMC simulations based on solid crystal structures. However, once the cages are dissolved in a solvent, entropy-driven filling by gas-phase species entails two thermodynamic hurdles: (1) solvent molecules must be expelled from the cage into the bulk liquid and (2) the incoming gas molecule must overcome the enthalpic penalty of leaving its solvated state to enter the cage. It may be useful in future treatments of these phenomena to consider the free energy associated with this transfer as possible descriptors, even though it may not be as straightforward to compute as the quantities we used in our screening approach. We emphasize that our TQMD simulations of solvated cages fully account for these effects, so this discussion does not point to a limitation of our detailed simulations of individual porous liquids.

Our spatial analysis reveals no evidence of liquid–liquid phase separation or large-scale aggregation of POCs in the 10 wt % chloroform-CC13 system, even under elevated pressures. However, the emergence of small cage clusters at higher pressures is a noteworthy observation. This subtle clustering behavior may still influence key macroscopic properties, such as viscosity, diffusivity, or rheological response under compression. Exploring how such pressure-dependent aggregation impacts the physical properties of porous liquids could provide valuable insights and become a potential direction for future work.

## Supplementary Material





## Notes

Figure S1: vapor–liquid coexistence curves for chloroform and DCM; Figure S2: isotherm of a pure and binary mixture of CO_2_ and chloroform in fugacity; Figure S3: isotherm of a pure and binary mixture of CO_2_ and DCM; Figure S4: isotherm of a binary mixture of CO_2_ and nine solvents; Figure S5: enthalpy of adsorption of different solvents at 273 K; Figure S6: parity plot of the linear regression model; Figure S7: comparison of simulated and experimental CO_2_ solubility in chloroform; Figure S8: comparison of simulated and experimental CO_2_ solubility in DME; Figure S9: trajectories of window-hopping chloroform molecules; Figure S10: schematic illustration of the two TQMD methods; Figure S11: evolution of density profile along the *z*-direction for TQMD Method 2; Figure S12: comparison of two TQMD methods in the CO_2_ isotherm; Figure S13: *Z*-direction density profiles from TQMD using three different solvents; Figure S14: 3D spatial density distributions of CC13 and CO_2_; and Table S1: average frequency of cluster occurrence (PDF)
Tabulated data (ZIP)

## References

[1] Fulvio P. F., Dai S. (2020). Porous Liquids: The Next Frontier. Chem.

[2] Egleston B. D., Mroz A., Jelfs K. E., Greenaway R. L. (2022). Porous
Liquids - the Future Is Looking Emptier. Chem
Sci.

[3] O’Reilly N., Giri N., James S. L. (2007). Porous Liquids. Chem.-Eur. J..

[4] Zhang J., Chai S.-H., Qiao Z.-A., Mahurin S. M., Chen J., Fang Y., Wan S., Nelson K., Zhang P., Dai S. (2015). Porous Liquids: A Promising
Class of Media for Gas Separation. Angew. Chem..

[5] Gaillac R., Pullumbi P., Beyer K. A., Chapman K., Keen D. A., Bennett T. D., Coudert F. X. (2017). Liquid Metal–Organic Frameworks. Nat. Mater..

[6] Ma L., Haynes C. J. E., Grommet A. B., Walczak A., Parkins C. C., Doherty C. M., Longley L., Tron A., Stefankiewicz A. R., Bennett T. D., Nitschke J. R. (2020). Coordination
Cages as Permanently
Porous Ionic Liquids. Nat Chem.

[7] Giri N., Del Pópolo M. G., Melaugh G., Greenaway R. L., Rätzke K., Koschine T., Pison L., Gomes M. F. C., Cooper A. I., James S. L. (2015). Liquids with Permanent Porosity. Nature.

[8] Zhu G., Liu Y., Flores L., Lee Z. R., Jones C. W., Dixon D. A., Sholl D. S., Lively R. P. (2018). Formation Mechanisms and Defect Engineering
of Imine-Based Porous Organic Cages. Chem. Mater..

[9] Rimsza J., Nenoff T. M. (2023). Design of Enhanced Porous Organic Cage Solubility in
Type 2 Porous Liquids. J. Mol. Liq..

[10] Kearsey R. J., Alston B. M., Briggs M. E., Greenaway R. L., Cooper A. I. (2019). Accelerated Robotic Discovery of
Type II Porous Liquids. Chem Sci.

[11] Zhang Z., Yang B., Zhang B., Cui M., Tang J., Qiao X. (2022). Type II Porous Ionic Liquid Based
on Metal-Organic Cages That Enables
l-Tryptophan Identification. Nat Commun.

[12] Deng Z., Ying W., Gong K., Zeng Y., Yan Y., Peng X. (2020). Facilitate Gas Transport
through Metal-Organic Polyhedra Constructed
Porous Liquid Membrane. Small.

[13] Lai B., Cahir J., Tsang M. Y., Jacquemin J., Rooney D., Murrer B., James S. L. (2021). Type 3
Porous Liquids
for the Separation of Ethane and Ethene. ACS
Appl Mater Interfaces.

[14] Cahir J., Tsang M. Y., Lai B., Hughes D., Alam M. A., Jacquemin J., Rooney D., James S. L. (2020). Type 3 Porous Liquids
Based on Non-Ionic Liquid Phases-a Broad and Tailorable Platform of
Selective. Fluid Gas Sorbents. Chem Sci.

[15] Hurlock M.
J., Lu L., Sarswat A., Chang C. W., Rimsza J. M., Sholl D. S., Lively R. P., Nenoff T. M. (2024). Exploitation of Pore Structure for
Increased CO_2_ Selectivity in Type 3 Porous Liquids. ACS Appl Mater Interfaces.

[16] Brand M. C., Rankin N., Cooper A. I., Greenaway R. L. (2023). Photoresponsive
Type III Porous Liquids. Chem. – Eur.
J..

[17] Liu S., Liu J., Hou X., Xu T., Tong J., Zhang J., Ye B., Liu B. (2018). Porous Liquid: A Stable ZIF-8 Colloid in Ionic Liquid
with Permanent Porosity. Langmuir.

[18] Chang C. W., Borne I., Lawler R. M., Yu Z., Jang S. S., Lively R. P., Sholl D. S. (2022). Accelerating Solvent
Selection for
Type II Porous Liquids. J. Am. Chem. Soc..

[19] Krishna R. (2019). Elucidation
and Characterization of Entropy Effects in Mixture Separations with
Micro-Porous Crystalline Adsorbents. Sep Purif
Technol.

[20] Krishna R., Van Baten J. M. (2020). Elucidation of Selectivity Reversals for Binary Mixture
Adsorption in Microporous Adsorbents. ACS Omega.

[21] Torres-Knoop A., Poursaeidesfahani A., Vlugt T. J. H., Dubbeldam D. (2017). Behavior of
the Enthalpy of Adsorption in Nanoporous Materials Close to Saturation
Conditions. J Chem Theory Comput.

[22] Poursaeidesfahani A., Torres-Knoop A., Rigutto M., Nair N., Dubbeldam D., Vlugt T. J. H. (2016). Computation of the Heat and Entropy of Adsorption in
Proximity of Inflection Points. Journal of Physical
Chemistry C.

[23] Krishna R., Smit B., Vlugt T. J. H. (1998). Sorption-Induced
Diffusion-Selective
Separation of Hydrocarbon Isomers Using Silicalite. J Phys Chem A.

[24] Rzepa C., Siderius D. W., Hatch H. W., Shen V. K., Rangarajan S., Mittal J. (2020). Computational Investigation
of Correlations in Adsorbate
Entropy for Pure-Silica Zeolite Adsorbents †. Journal of Physical Chemistry C.

[25] Dauenhauer P. J., Abdelrahman O. A. (2018). A Universal
Descriptor for the Entropy of Adsorbed
Molecules in Confined Spaces. ACS Cent Sci.

[26] Hasell T., Culshaw J. L., Chong S. Y., Schmidtmann M., Little M. A., Jelfs K. E., Pyzer-Knapp E. O., Shepherd H., Adams D. J., Day G. M. (2014). Controlling
the Crystallization of Porous Organic Cages: Molecular Analogs of
Isoreticular Frameworks Using Shape-Specific Directing Solvents. J. Am. Chem. Soc..

[27] Borne I., Saigal K., Jones C. W., Lively R. P. (2023). Thermodynamic Evidence
for Type II Porous Liquids. Ind. Eng. Chem.
Res..

[28] Rimsza J. M., Nenoff T. M. (2024). Critical Role of Solvation on CC13
Porous Organic Cages
for Design of Porous Liquids. J. Mol. Liq..

[29] Egleston B., Luzyanin K. V, Brand M. C., Clowes R., Briggs M. E., Greenaway R. L., Cooper A. I. (2020). Controlling Gas Selectivity in Molecular
Porous Liquids by Tuning the Cage Window Size. Angew. Chem., Int. Ed..

[30] Jorgensen W. L., Maxwell D. S., Tirado-Rives J. (1996). Development and Testing of the OPLS
All-Atom Force Field on Conformational Energetics and Properties of
Organic Liquids. J. Am. Chem. Soc..

[31] Eggimann B.
L., Sunnarborg A. J., Stern H. D., Bliss A. P., Siepmann J. I. (2014). An Online
Parameter and Property Database for the TraPPE Force Field. Mol Simul.

[32] Kamath G., Georgiev G., Potoff J. J. (2005). Molecular
Modeling of Phase Behavior
and Microstructure of Acetone-Chloroform-Methanol Binary Mixtures. Journal of Physical Chemistry B.

[33] Wang J., Wolf R. M., Caldwell J. W., Kollman P. A., Case D. A. (2004). Development
and Testing of a General Amber Force Field. J. Comput. Chem..

[34] Kresse G., Furthmüller J. (1996). Efficiency
of Ab-Initio Total Energy Calculations for
Metals and Semiconductors Using a Plane-Wave Basis Set. Comput. Mater. Sci..

[35] Dubbeldam D., Calero S., Ellis D. E., Snurr R. Q. (2016). RASPA: Molecular
Simulation Software for Adsorption and Diffusion in Flexible Nanoporous
Materials. Mol Simul.

[36] Lu L., Chang C. W., Schuyten S., Roy A., Sholl D. S., Lively R. P. (2025). Nonadditive CO_2_ Uptake
of Type II Porous
Liquids Based on Imine Cages. ChemPhysChem.

[37] Martínez-Veracoechea F., Muller E. A. (2005). Temperature-Quench Molecular Dynamics Simulations for
Fluid Phase Equilibria. Mol Simul.

[38] Gelb L. D., Müller E. A. (2002). Location
of Phase Equilibria by Temperature-Quench
Molecular Dynamics Simulations. Fluid Phase
Equilib..

[39] Linstrom P. J., Mallard W. G. (2001). The NIST Chemistry WebBook: A Chemical Data Resource
on the Internet. J. Chem. Eng. Data.

[40] Shirono K., Morimatsu T., Takemura F. (2008). Gas Solubilities (CO_2_,
O_2_, Ar, N_2_, H_2_, and He) in Liquid
Chlorinated Methanes. J. Chem. Eng. Data.

[41] Xu M., Wang S., Xu L. (2019). Screening
of Physical-Chemical Biphasic
Solvents for CO_2_ Absorption. International
Journal of Greenhouse Gas Control.

[42] Gómez-Díaz D., Parajó M., Richoux O., La Rubia M. D., Rumbo A. (2021). Kinetics,
Absorption and Regeneration of Biphasic Solvent with Ethylpiperidine
for Carbon Dioxide Absorption. Fuel.

[43] Liu X., Niu X., Zhan G., Xing L., Huang Z., Yuan B., Peng Y., Chen Z., Li J. (2024). Dynamic Phase-Splitting
Behaviour of Biphasic Solvent for Carbon Capture in a Novel Annular
Phase Separator. Appl Energy.

[44] Ester, M. ; Kriegel, H.-P. ; Sander, J. ; Xu, X. A Density-Based Algorithm for Discovering Clusters in Large Spatial Databases with Noise. In Proceedings of the Second International Conference on Knowledge Discovery and Data Mining; AAAI Press, 1996; pp 226–231.

