# Supplementary material for: Entropy-Driven Porous Liquids Allowing Gas Solubility in Solvent-Filled Imine-Based Porous Organic Cages
Source: J Phys Chem B. 2025 Aug 6;129(33):8501–8. doi: 10.1021/acs.jpcb.5c04176 (PMC12376116; doi:10.1021/acs.jpcb.5c04176)
Supplement: Supplementary file 1 [file jp5c04176_si_001.pdf]

Supplementary information for

Entropy-driven Porous Liquids Allowing Gas Solubility in Solvent Filled Imine-based Porous Organic Cages

Chao-Wen Chang[a], and David S. Sholl[b*]

[a]School of Chemical & Biomolecular Engineering, Georgia Institute of Technology, Atlanta, GA 30332, USA

[b]Oak Ridge National Laboratory, Oak Ridge, TN 37839, USA

Corresponding Author: shollds@ornl.gov

Table of Contents

**Figure S1**	Vapor−liquid coexistence curves for chloroform and dichloromethane	S2
**Figure S2**	Isotherm of pure and binary mixture of CO_2 and chloroform in fugacity	S3
**Figure S3**	Isotherm of pure and binary mixture of CO_2 and dichloromethane	S4
**Figure S4**	Isotherm of binary mixture of CO_2 and nine solvents	S5
**Figure S5**	Enthalpy of adsorption of different solvents at 273K	S6
**Figure S6**	Parity plot of linear regression model	S7
**Figure S7**	Comparison of simulated and experimental CO_2 solubility in chloroform	S8
**Figure S8**	Comparison of simulated and experimental CO_2 solubility in 1,2-dimethoxyethane	S8
**Figure S9**	The trajectories of window-hopping chloroform molecules	S9
**Figure S10**	Schematic illustration of the two TQMD methods	S10
**Figure S11**	Evolution of density profile along the z-direction for TQMD Method 2	S11
**Figure S12**	Comparison of two TQMD methods in CO_2 isotherm	S12
**Figure S13**	Z-direction density profiles from TQMD using three different solvents	S13
**Figure S14**	3D spatial density distributions of CC13 and CO_2	S14
**Table S1**	The average frequency of cluster occurrence	S15
	References	S16

A compressed zip archive accompanies this Supplementary Information document. It contains three parts:

1. **sample input file/binaryGCMC/** – Sample RASPA2 input files (simulation.input, force_field_mixing_rules.def, etc.) used for the grand-canonical Monte-Carlo (GCMC) calculations of CO_2 sorption and selectivity.

2. **sample input file/TQMD/** – Sample LAMMPS input files (.in and .mol files) for the Temperature Quench Molecular Dynamics (TQMD) simulations discussed in the main text.

3. **SI_Tabulated_Data.xlsx/** – An excel spreadsheet that compiles numerical values plotted in the manuscript (Figures 1–3, Tables 1).

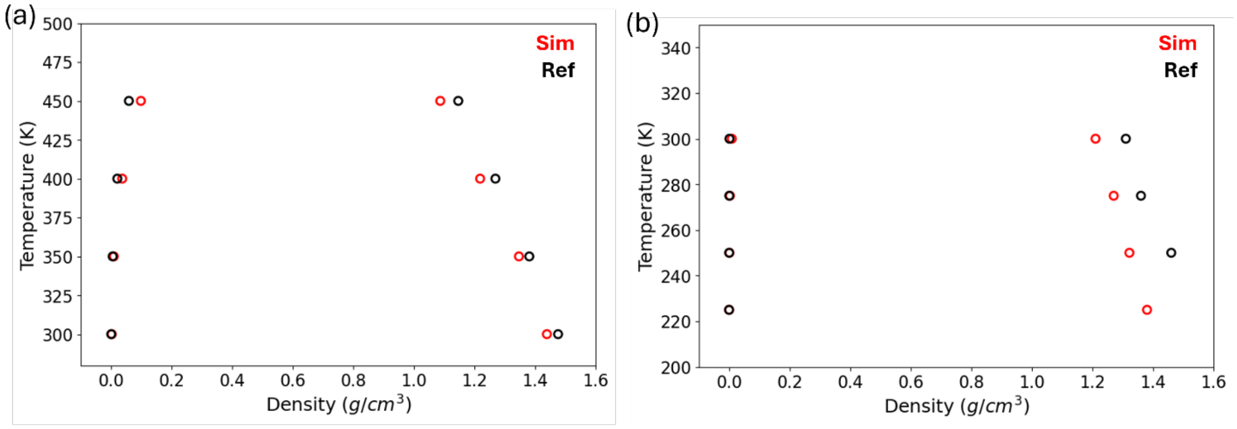

Figure S1. Vapor–liquid coexistence curves for (a) chloroform and (b) dichloromethane, with molecular simulation data shown as red points using TQMD and experimental data from Kamath et al.[1] and the NIST database.

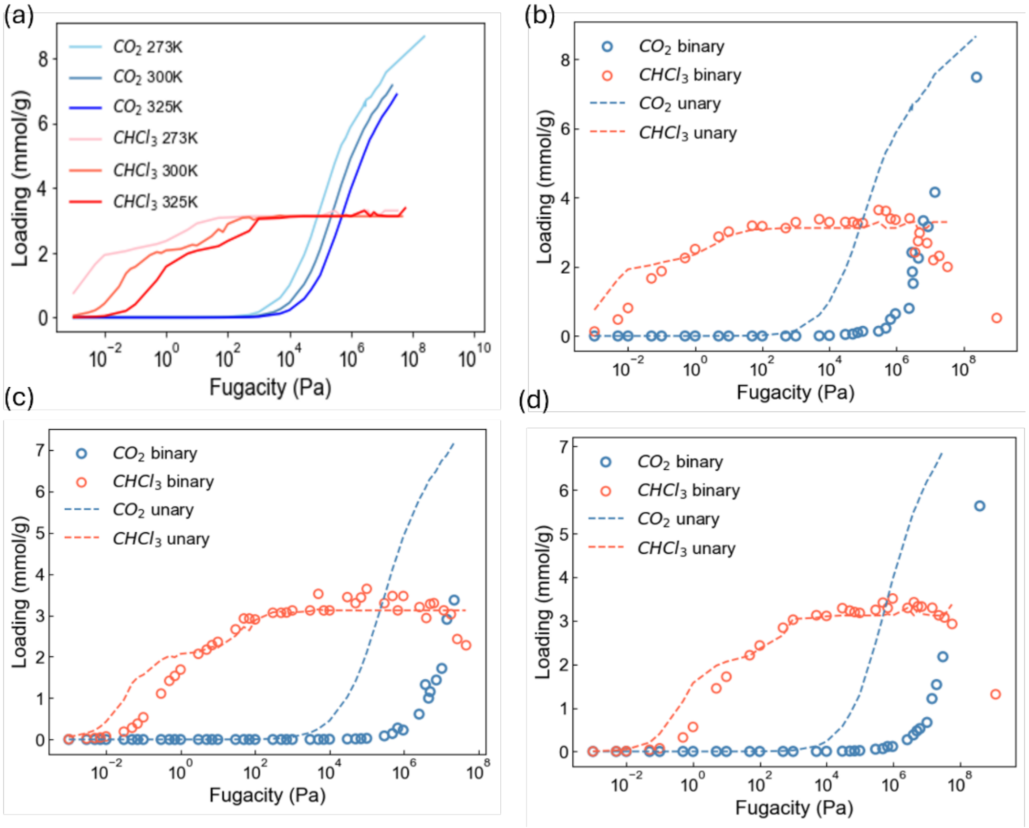

Figure S2. Same data as in Figure 1, except fugacity (Pa) is used on the x-axis instead of pressure. Fugacity values were calculated using the Peng–Robinson equation of state. (a) GCMC data for single-component adsorption isotherms of CO_2 and chloroform in CC13α crystal at 273 K, 300 K, and 325 K. (b-d) GCMC data for binary-component adsorption isotherms of CO_2 and chloroform in CC13 crystal at (b) 273 K, (c) 300 K, and (d) 325 K. Dashed lines represent the single-component isotherms of CO_2 and chloroform.

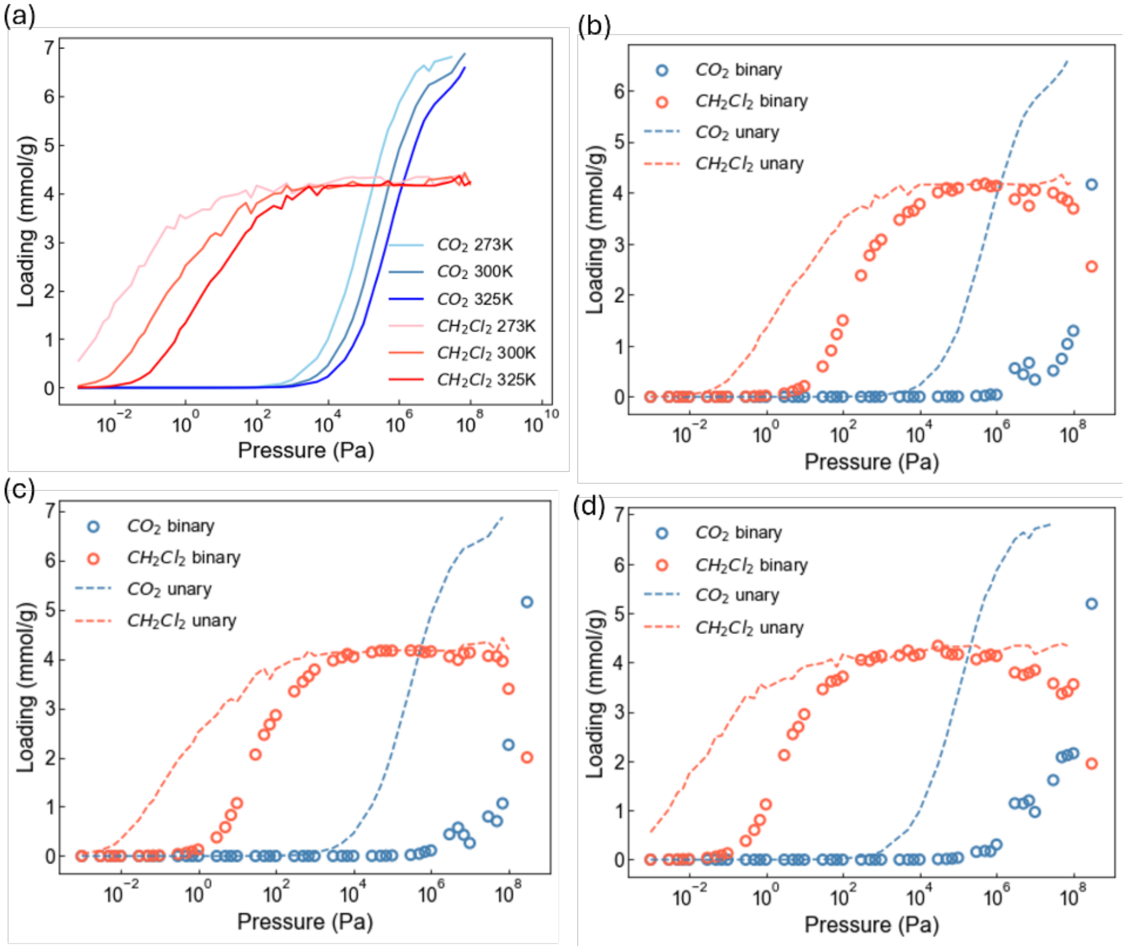

Figure S3. (a) GCMC data for single-component adsorption isotherms of CO_2 and dichloromethane in a rigid CC13α crystal at 273, 300, and 325 K. (b-d) GCMC data for binary-component adsorption isotherms of equimolar CO_2 and dichloromethane in a rigid CC13α crystal at (b) 273 K, (c) 300 K, and (d) 325 K. Dashed lines represent the single-component isotherms of CO_2 and chloroform.

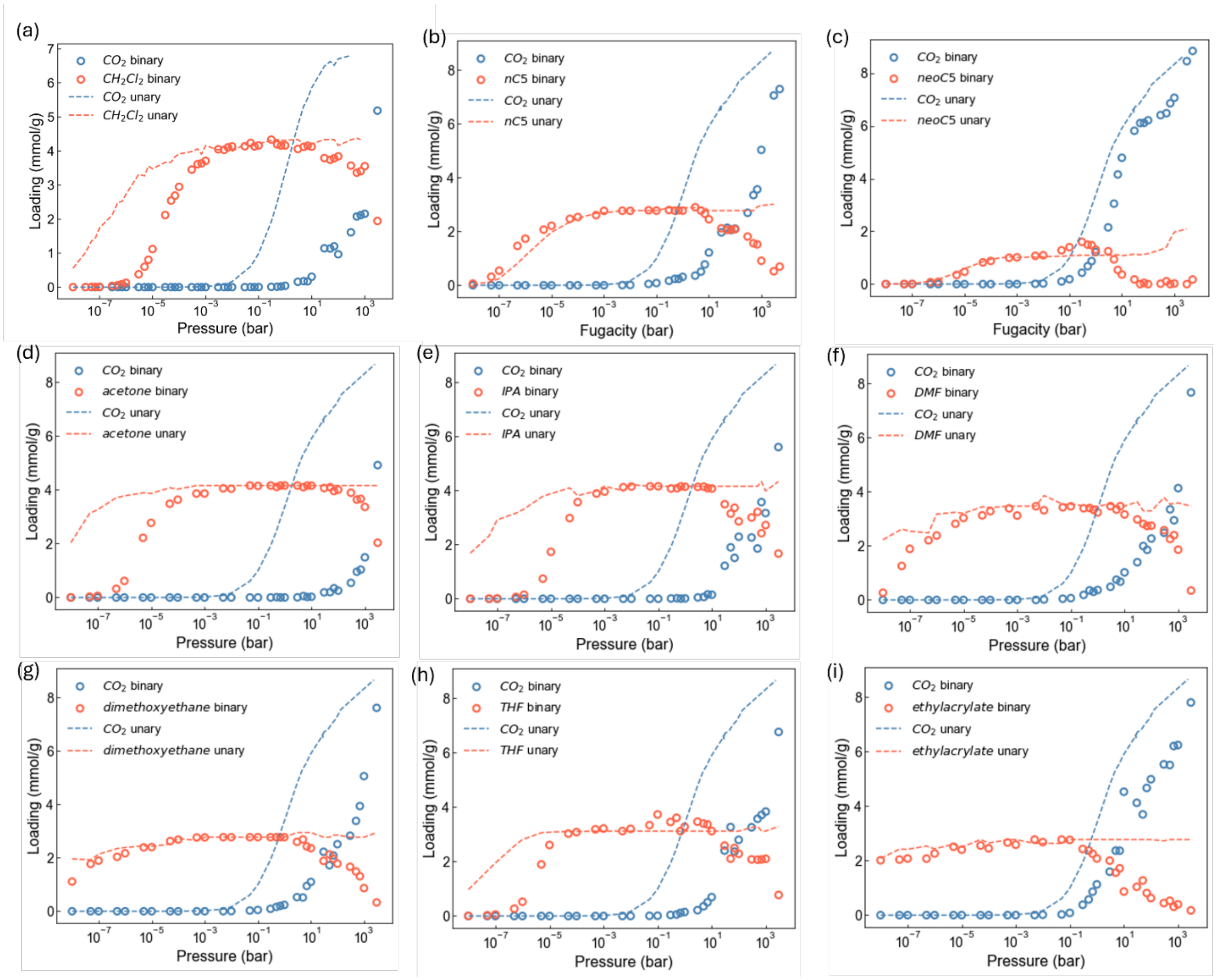

Figure S4. GCMC data for binary-component adsorption isotherms of equimolar CO_2 and selected solvent, namely (a) dichloromethane, (b) n-pentane, (c) neopentane, (d) acetone, (e) isopropyl alcohol (IPA), (f) N,N-dimethylformamide (DMF), (g) dimethoxyethane (DME), (h) tetrahydrofuran (THF), and (i) ethyl acrylate in a rigid CC13α crystal at 273 K. Dashed lines represent the single-component isotherms of CO_2 and chloroform.

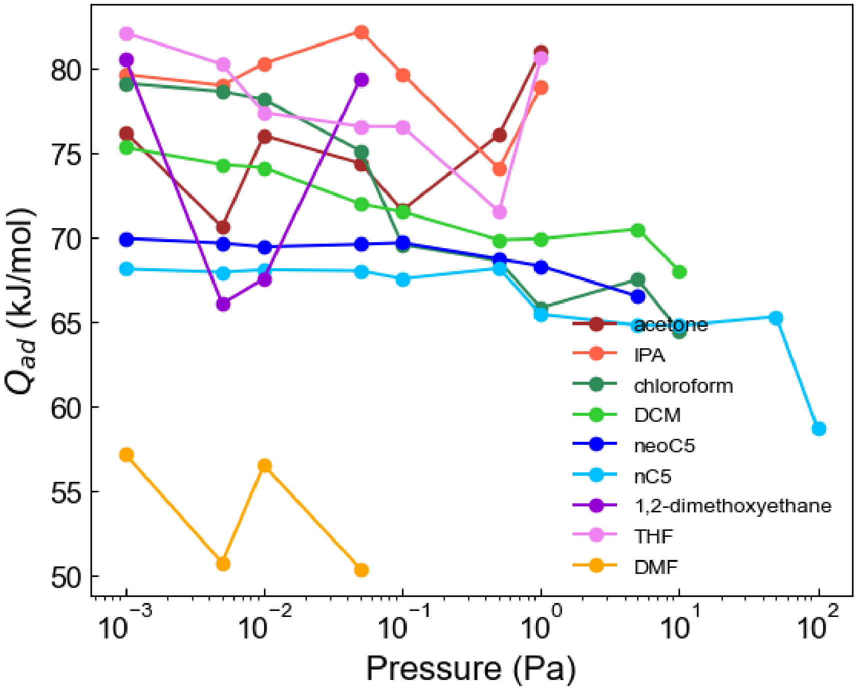

Figure S5. Enthalpy of adsorption at 273K of different solvents in CC13α. Equilibrium adsorption of porous material can be predicted via molecular simulations using Grand Canonical Monte Carlo (GCMC) through RASPA code. Enthalpy of adsorption is only calculated before saturation.

Index	Solvent Species
0	neo-pentane
1	ethyl acrylate
2	dimethoxyethane
3	tetrahydrofuran
4	n-pentane
5	N,N-dimethylformamide
6	chloroform
7	isopropyl alcohol
8	dichloromethane
9	acetone

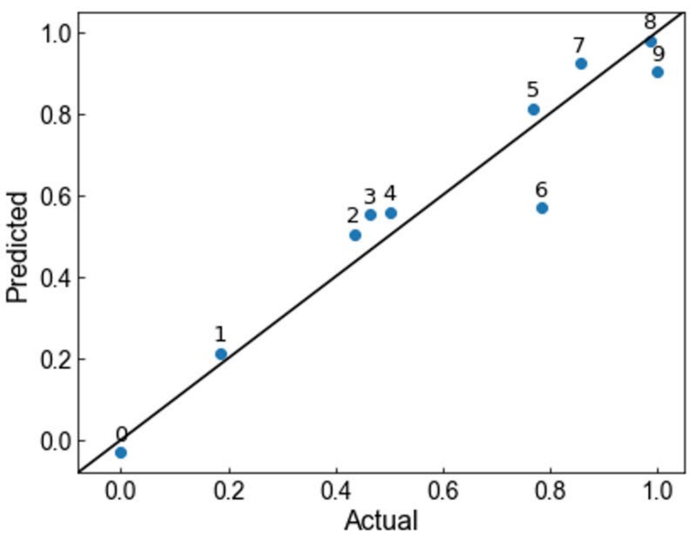

Figure S6. Based on Table 1, we normalize ln (P_{rev}), Δq_{sat} and ΔQ_{st}^{0} from 0 to 1 and then perform multi linear regression (MLR) to fit ln (P_{rev}) based on Δq_{sat} and ΔQ_{st}^{0}. We then obtain the model fitting equation ln $(P_{rev}) = 0.008 + \mathbf{1.04}\,\Delta q_{sat} - \mathbf{0.38}\,\Delta Q_{st}^{0}$ with $r^2 = 0.92$. The figure is the parity plot between the actual ln (P_{rev}) value and the MLR model.

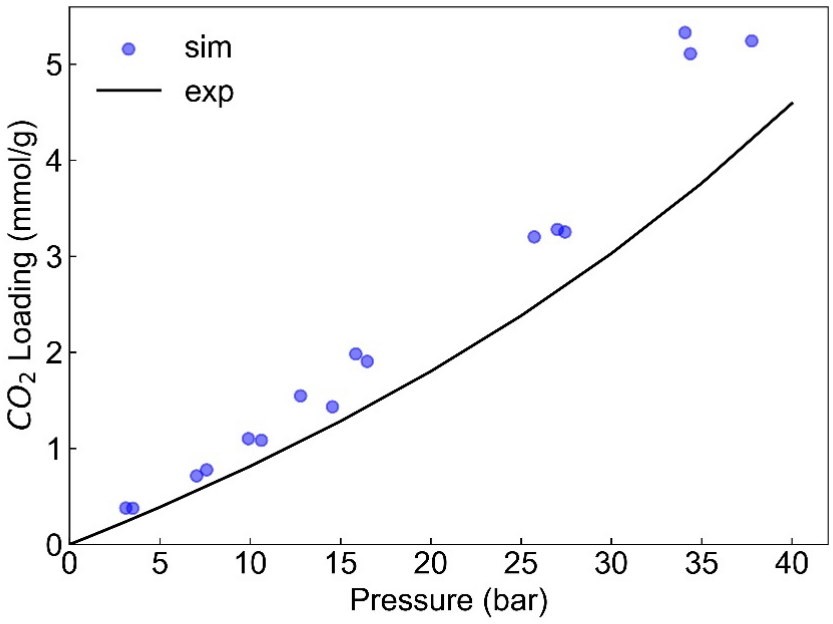

Figure S7. Solubility of CO_2 in chloroform at 300K obtained by TQMD and the experimental data of Shirono et al.[2]

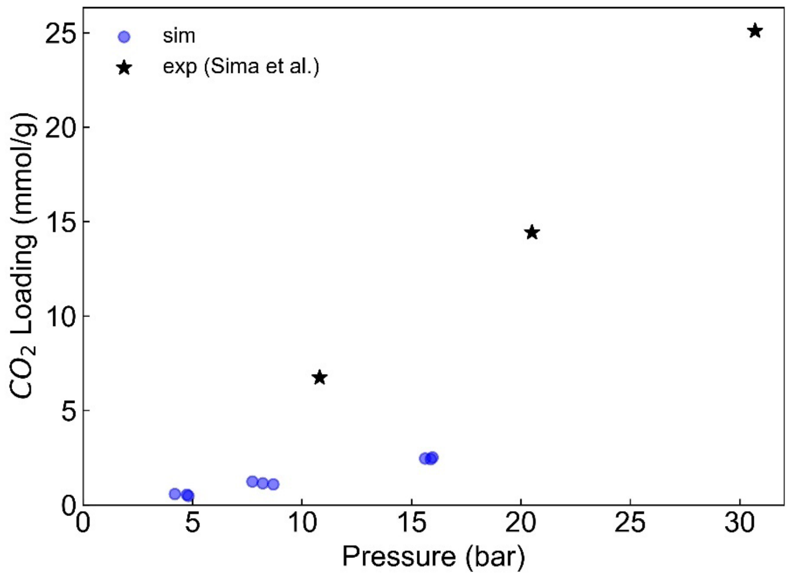

Figure S8. Solubility of CO_2 in 1,2-dimethoxyethane (DME) at 313K obtained by TQMD and the experimental data of Sima et al.[3]

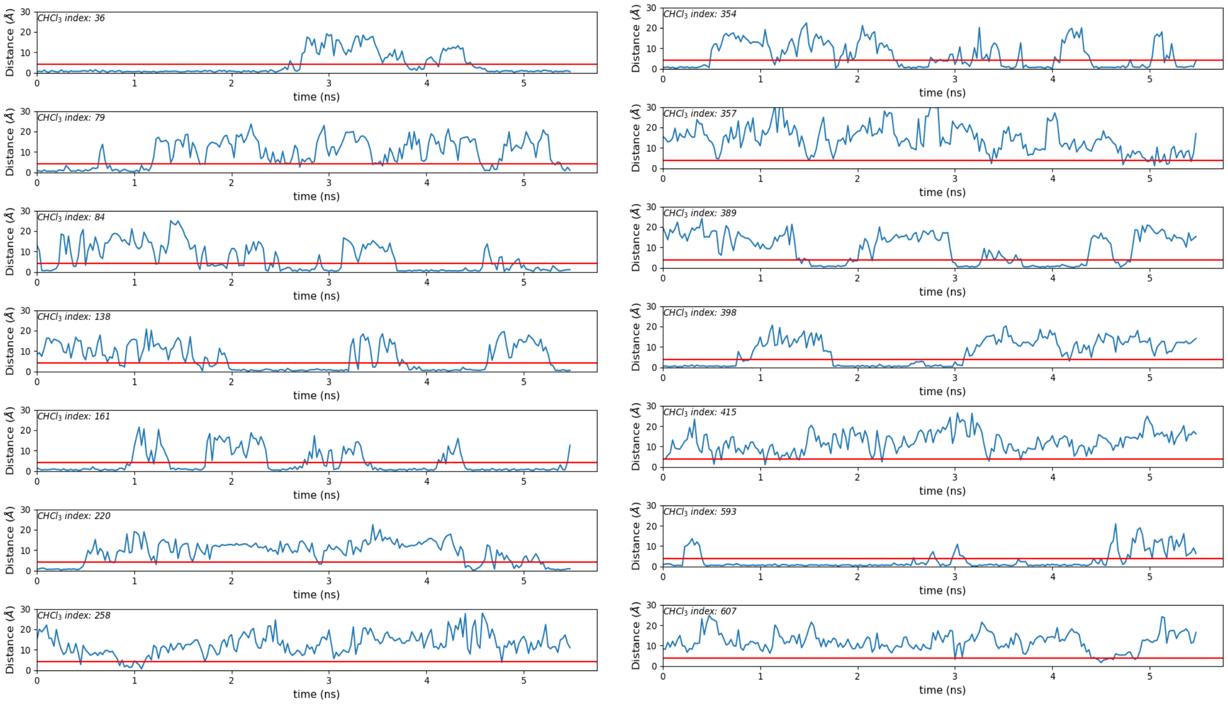

Figure S9. The trajectories of 14 only window-hopping chloroform molecules (within a system comprising 720 chloroform molecules and 10 cages) were tracked as they transitioned between the interior and exterior of the cages over a 5.5 ns simulation period at 300 K and 1 bar. The vertical axis is the distance to the closest cage's center of mass. The red line in each plot is the boundary of inside/outside of the cage.

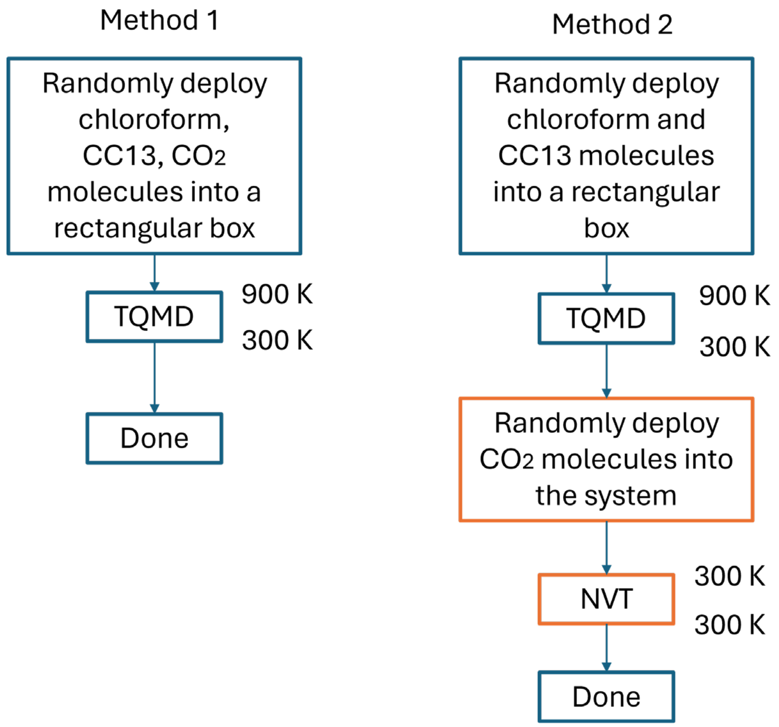

Figure S10. Schematics for performing TQMD of chlroform-CC13 mixture with CO_2 at 300K. Method 1 deploys all three components simultaneously, whereas Method 2 first introduces chloroform and CC13. After the system establishes distinct liquid and gas phases, CO_2 is then introduced into the gas phase.

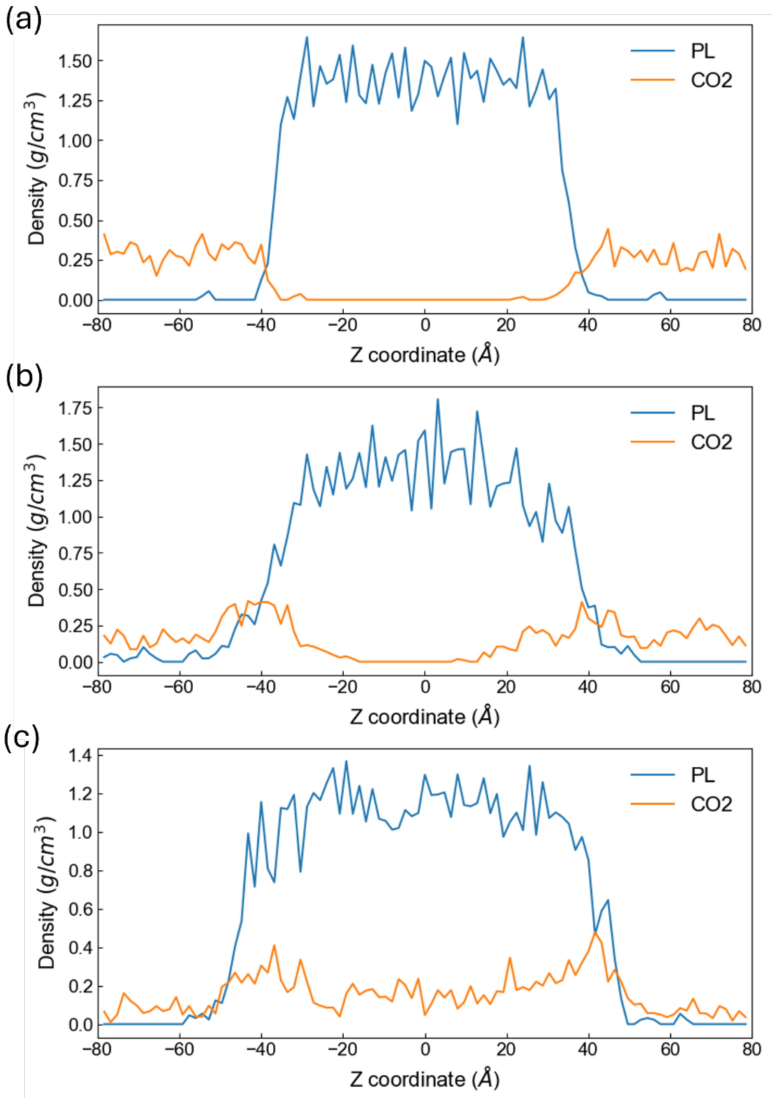

Figure S11. An example of the evolution of the density profile along the z-direction for TQMD Method 2 (presented in **Figure S10**) at 300K. The porous liquid system consists of 720 chloroform molecules and 10 cages. CO_2 is initially deployed in the gas phase, with the density profile shown at (a) time zero. Snapshots of the density profile are provided for (b) 10 ps and (c) 250 ps, illustrating the evolution of the system over time.

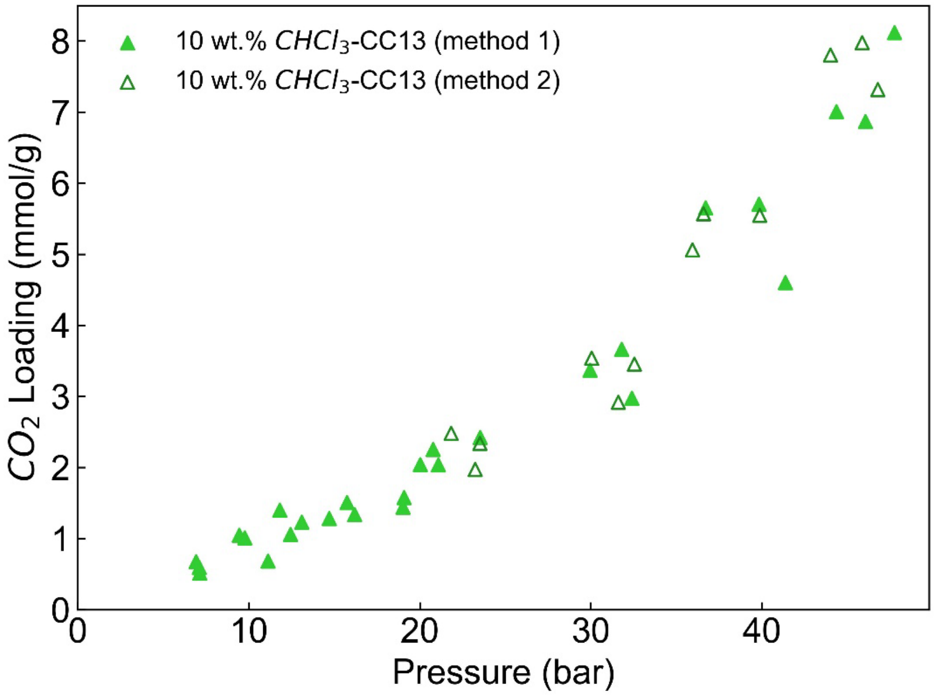

Figure S12. CO_2 solubilities in 10 wt.% chloroform-CC13 at 300K using the two methods of initializing molecules deploying described in **Figure S10.**

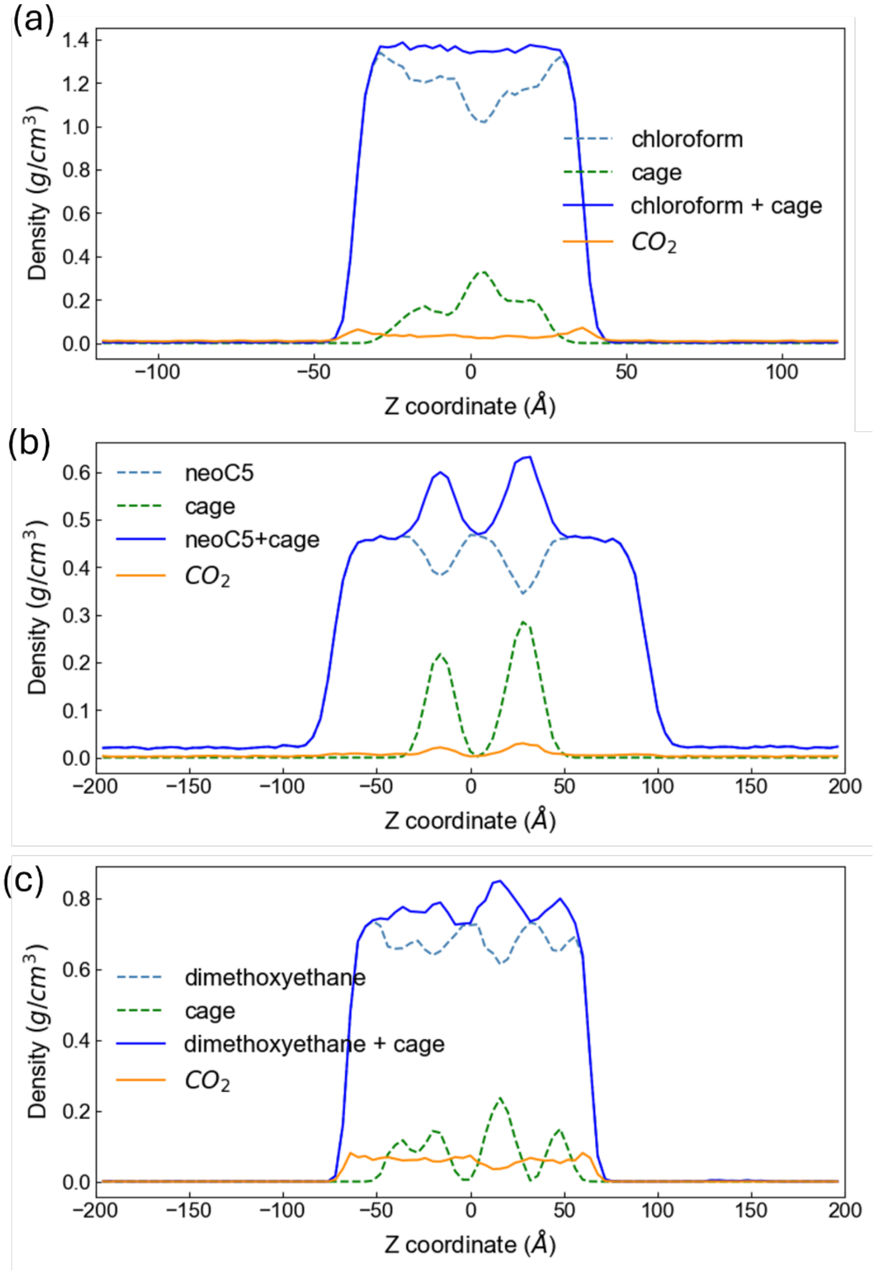

Figure S13. Time-average density profiles along the z-direction in TQMD simulations using (a) chloroform, (b) neo-pentane, and (c) dimethoxyethane as solvents, in a system comprising 10 CC13 molecules, 100 CO_2 molecules, and a number of solvent molecules determined to achieve a 10 wt% mixture.

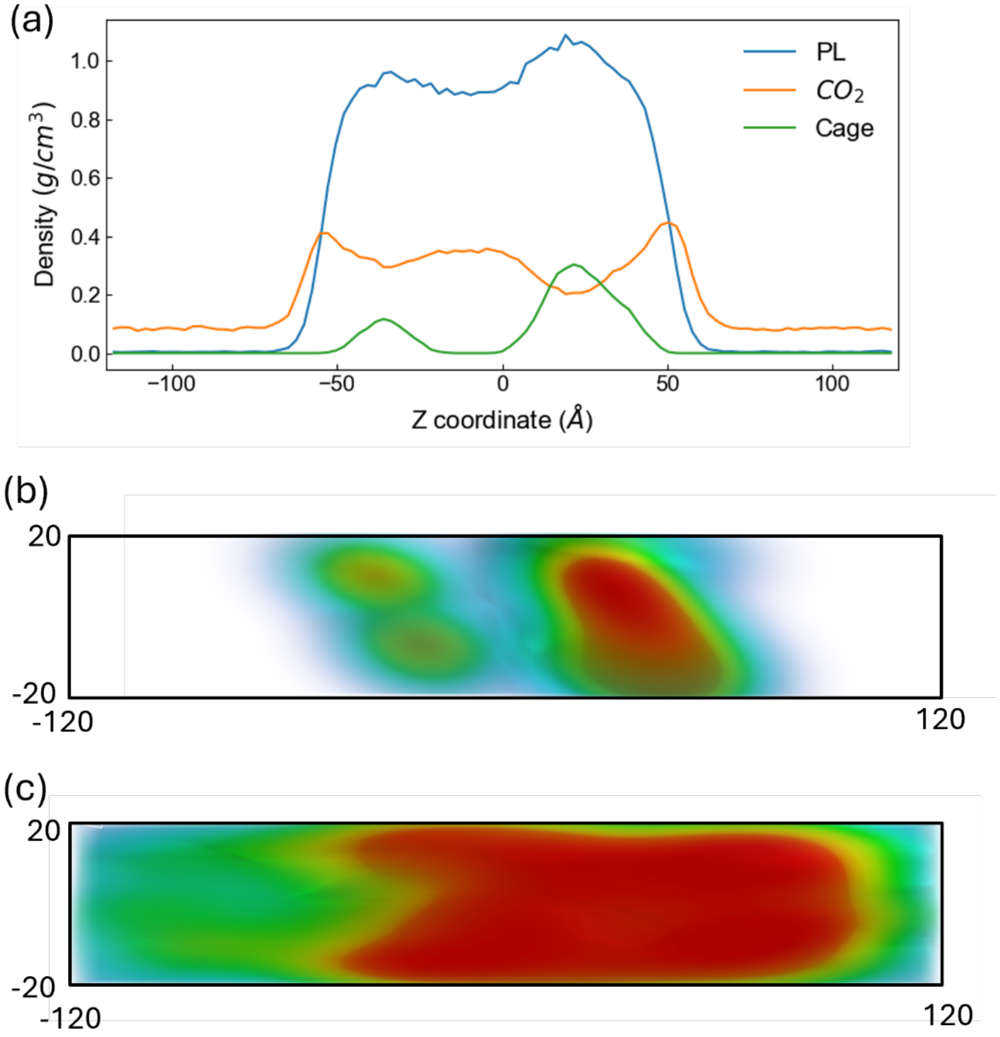

Figure S14. For the 10 wt.% chloroform–CC13 system simulated using TQMD at 300 K, the pressure was determined to be 46 bar. (a) The time-averaged density profile along the z-direction was computed over 100 snapshots spanning 2.5 ns. (b) and (c) show the 3D spatial density distributions of CC13 cages and CO_2, respectively, viewed perpendicular to the xz-plane. Regions with redder coloration indicate higher local densities.

Table S1. The average frequency of cluster occurrence with a specific number of cages under varying pressures was analyzed using the DBSCAN algorithm, with the number of neighbors set to 2 and a radius threshold of 14.3 Å. The simulation system consisted of a 10 wt.% CC13-chloroform solution containing 40 CC13 cages with saturated CO_2 loading.

	# cage in a cluster	2	3	4	5	6	7	8	9	10	11	12
7 bar	run # 1	3.48	1.23	1.15	0.295	0.145	0.03	0.005	0	0	0	0
	run # 2	4.13	1.49	0.41	0.21	0.005	0	0	0	0	0	0
	run # 3	4.395	1.325	0.55	0.21	0.01	0	0	0	0	0	0
12 bar	run # 1	5.185	0.695	0.21	0.235	0.19	0.24	0.07	0	0	0	0
	run # 2	5.145	1.295	0.37	0.185	0	0.005	0	0	0	0	0
	run # 3	4.505	1.435	0.455	0.395	0.19	0.01	0	0	0	0	0
22 bar	run # 1	4.395	1.345	0.49	0.155	0.06	0.01	0.04	0	0	0	0
	run # 2	4.385	1.255	0.415	0.135	0.015	0	0	0	0	0	0
	run # 3	4.945	1.045	0.41	0.035	0.015	0	0	0	0	0	0
31 bar	run # 1	4.465	1.585	0.375	0.12	0.06	0.11	0.04	0.02	0.02	0	0
	run # 2	4.865	1.175	0.25	0.115	0.03	0	0	0	0	0	0
	run # 3	5.005	1.47	0.39	0.09	0.04	0	0.015	0	0	0	0
39 bar	run # 1	5.57	1.395	0.445	0.12	0.025	0	0	0	0	0	0
	run # 2	4.695	2.08	0.865	0.28	0.095	0.005	0.01	0.005	0	0	0
	run # 3	5.47	1.34	0.485	0.205	0.035	0.005	0	0	0	0	0
46 bar	run # 1	5.805	1.9	0.61	0.225	0.045	0.01	0	0	0	0	0
	run # 2	4.77	1.535	0.405	0.4	0.19	0.14	0	0.015	0.02	0.01	0.005
	run # 3	3.59	1.135	0.415	0.285	0.2	0.06	0.015	0.005	0	0	0

References

(1) Kamath, G.; Georgiev, G.; Potoff, J. J. Molecular Modeling of Phase Behavior and Microstructure of Acetone-Chloroform-Methanol Binary Mixtures. *Journal of Physical Chemistry B* **2005**, *109* (41), 19463–19473. https://doi.org/10.1021/jp0535238.

(2) Shirono, K.; Morimatsu, T.; Takemura, F. Gas Solubilities (CO_2, O_2, Ar, N_2, H_2, and He) in Liquid Chlorinated Methanes. *J Chem Eng Data* **2008**, *53* (8), 1867–1871. https://doi.org/10.1021/je800200j.

(3) Sima, S.; Secuianu, C.; Feroiu, V. Phase Equilibria of CO_2 + 1,2-Dimethoxyethane at High-Pressures. *Fluid Phase Equilib* **2018**, *458*, 47–57. https://doi.org/10.1016/j.fluid.2017.11.008.
